# The “EU chemicals strategy for sustainability” questions regulatory toxicology as we know it: is it all rooted in sound scientific evidence?

**DOI:** 10.1007/s00204-021-03091-3

**Published:** 2021-06-22

**Authors:** Matthias Herzler, Philip Marx-Stoelting, Ralph Pirow, Christian Riebeling, Andreas Luch, Tewes Tralau, Tanja Schwerdtle, Andreas Hensel

**Affiliations:** German Federal Institute for Risk Assessment (BfR), Berlin, Germany

**Keywords:** Chemicals strategy for sustainability, European Commission, Evidence-based risk assessment, Regulatory toxicology

## Introduction

Over the past decades, there has been an unprecedented regulatory drive in public health protection in the European Union, making large-scale toxicological incidents or mass poisonings such as, e.g. the thalidomide disaster of the 1950s–60s or the Seveso incident in 1976 an issue of the past. Although this is rarely perceived by the media and the general public, the implementation of a complex and interdependent network of regulations for chemical substances, including industrial chemicals, plant protection products, biocides, or chemicals in food and feed has minimised toxicological risks and has continuously increased public health and wellbeing in the EU. Moreover, although this framework already provides one of the most advanced regulatory systems worldwide, it is constantly pressed for improvement by scientific progress as well as an ever-increasing public awareness. From the perspective of a governmental authority in charge of consumer protection, this continuous evolutionary process is in fact key for safeguarding that consumers are protected according to state-of-the-art scientific knowledge and that the respective regulatory processes are optimised with respect to both their efficiency and effectiveness.

In December 2019, the European Commission (COM) announced their political vision for sustainability, the “European Green Deal” (European Commission 2019). As part of this vision, the COM called for “a zero pollution ambition for a toxic-free environment”. In October 2020, this aspect was further elaborated in the COM’s “Chemicals Strategy for Sustainability Towards a Toxic-Free Environment” (the “CSS”, as it is now commonly known), accompanied by an action plan and so-called “Staff Working Documents” (SWDs) on selected aspects of the CSS (European Commission 2020b; c, d, e, f, g). With these publications, the COM has started an implementation process of remarkable speed and ambition, in particular when considering that until now, with the exception of a few public consultations on very general roadmaps (European Commission 2020a,2021a; e), the COM has sought scientific input almost exclusively from its own services and agencies.

The complex scientific, economical, and societal questions related to the CSS demand a broad discussion with all parties involved in, or affected by, the regulatory risk assessment of chemicals in the EU. Recently, the COM installed a so-called “High-Level Roundtable” (HRT) on the implementation of the CSS composed of industry representatives, NGOs, academic institutions, and international organisations with the mission “*to realise the objectives of the Chemicals Strategy and to monitor its implementation in dialogue with the stakeholders concerned. Discussions will focus in particular on how to support the transition to safe and sustainable chemicals and to a toxic-free environment*” (European Commission 2021b). It will be key to the success of the CSS that the parties represented in the HRT establish a common high-level (sic!) understanding of the main measures needed to improve consumer and environmental health and to streamline the regulatory processes. Nevertheless, it is also obvious that the HRT cannot be the forum where the many and complex scientific questions surrounding the CSS are discussed in-depth, all the more so, since the EU Member States (MS) are not represented in the HRT beyond the one MS currently holding the presidency over the Council (rotating every 6 months).

However, the nature and number of the open scientific questions surrounding the CSS demand a much broader as well as deeper scientific discussion to achieve its goals of sustainability and improved consumer health protection in a scientifically adequate, appropriate, and resource-efficient way. In particular, this process should involve those MS scientific institutions, which currently perform the major share of the European Union’s risk assessment groundwork on industrial chemicals and product safety. Many of these institutions, like the German Federal Institute for Risk Assessment (BfR), have a decade-long record of both practically applying and further developing the principles and methods of the science of regulatory risk assessment for consumer health protection in the EU. As both, renowned research and regulatory institutions, it is one of their foremost tasks to safeguard that scientific rigour is applied as the cornerstone of chemical risk assessment, to allow for unbiased and proportionate risk management by the risk managers and policy makers. In addition, MS risk assessment authorities are deeply rooted in the international risk assessment community, being at the forefront of progressing risk assessment methodology both in terms of research and practical implementation at the international level, e.g. under the umbrella of the Organization for Economic Co-Operation and Development (OECD) or the World Health Organization (WHO).

In the view of the authors of this commentary, it is exactly this expertise, which is needed to make the strategy a success by helping to avoid potential failure due to inherent scientific shortcomings:

1. The justification for the measures proposed by the CSS often lacks rigour with respect to the critical, systematic, and unbiased review of the available scientific evidence required for such a project. This not only inappropriately belittles the effectiveness^1^ of the current system of chemicals legislation, it also bears the risk of strong interpretational bias. At best, such an approach is likely to face subsequent scientific scrutiny and the need for tedious readjustments; at worst, it runs the risk of merely amplifying arbitrary concerns instead of following an evidence-based approach to focus the overall limited resources on pressing issues that matter (see “Is the current system effective enough to protect EU citizens sufficiently against inadequately controlled risks arising from exposure to chemicals?“).
2. The CSS and its associated documents clearly aim at improving the efficiency^2^ of the existing system. In light of the often painstakingly long time currently needed to achieve regulation of substances of concern, this initiative is highly welcome. Nevertheless, this delay is most prominently caused by the frequent lack of crucial information to perform reliable risk assessment and by the slow and tedious administrative procedures involved (European Commission 2018). There is an unarguable need for improving data quality and availability as well as to significantly shorten and streamline the respective administrative and legal procedures. It appears nevertheless questionable whether proposed approaches such as, e.g. the hazard-based “generic approach to risk management” or the “Mixture Assessment Factor” (MAF) can serve these purposes. On the contrary, the more “pragmatic” but at the same time less scientifically sound such proposals are, the more can they be expected to create conceptual incompatibilities and severe problems for downstream regulation later on. In the end, this might, therefore, do more harm than good (see “Measures proposed by the COM to improve the efficiency of the current system”).

For the sake of space and readability, this commentary will focus on key aspects of consumer health protection in the context of chemical and product safety. Aspects of occupational or environmental health (e.g. general air pollution from energy production, industrial processes, or traffic), or ecotoxicity as such, will not be touched upon. The same holds for other important elements of the CSS, such as the production of “safe and sustainable by design” chemicals or the circular economy, which are nevertheless acknowledged as important issues of the future. It is also explicitly noted that the views expressed herein are exclusively those of the BfR and not (necessarily) those of other German governmental agencies or ministries.

## Is the current system effective enough to protect EU citizens sufficiently against inadequately controlled risks arising from exposure to chemicals?

### A bird’s-eye view on the level of protection of the EU population against chemical risk

Risk assessment for consumer safety in all major EU chemical legislation systems aims at safeguarding the health of the general population, including sensitive sub-populations such as children or the elderly. The CSS itself acknowledges that the EU “*already has one of the most comprehensive and protective regulatory frameworks for chemicals, supported by the most advanced knowledge base globally. This regulatory framework is increasingly becoming a model for safety standards worldwide. The EU has been undeniably successful in creating an efficiently functioning internal market for chemicals, in reducing the risks to humans and the environment posed by certain hazardous chemicals, such as carcinogens and heavy metals, and in providing a predictable legislative framework for companies to operate in*.” (European Commission 2020g, p. 1). Inter alia, the various overarching and sector-specific regulations (e.g. REACH or the Plant Protection Product and Biocidal Product Regulations) address this using safety factors, sub-population-specific reference and exposure values, or adapted exposure scenarios.

Still, in various places, the CSS highlights a necessity for further improving the protection of “vulnerable groups”. However, without a detailed assessment of which risks are currently deemed to be insufficiently addressed, it is hard to establish whether additional regulation might be necessary or existing regulation might need to be improved, and for which part of the population. On p. 4, the CSS goes so far as to state a need to “*restore human health and environment to a good quality status*” with respect to “*substances of concern*”, thereby insinuating that this quality is currently not “good”. This not only contrasts the above appraisal of the overall status of protection of the general population in the EU against chemical risks; also, the CSS does not present further evidence in support of that claim.

Without doubt, the overall level of protection of human health in the EU against possible risks arising from the exposure to chemicals is high, in particular when compared to other regions of the world. Neither Eurostat statistics on life expectancy in the EU (European Commission 2021d), nor healthy life year statistics (European Commission 2021c) give rise to particular concern that—in the EU—chemical risk is an important, or even growing, detrimental factor to human health (aside from self-intoxication by smoking, drinking alcohol or drug abuse, which are not the subject of this commentary). On the contrary, both endpoints (i.e. life expectancy and healthy life years), while varying between EU Member States, have been rising more or less constantly for many years. Excluding genetic anomalies, and not corrected for the increase of parental age at birth, the prevalence of anomalies in the newborn in the EU has been practically constant (at around 200/10,000) for the past 40 years.^3^ Moreover, the fact that the world population has almost tripled since 1950 (with even faster population growth in areas of the world commanding only little or no chemical control at all) does not seem to point at a fundamental reproduction crisis of mankind.

The CSS prominently and repeatedly justifies the need for action by referring to public concern: “*84% of Europeans are worried about the impact of chemicals present in everyday products on their health, and 90% are worried about their impact on the environment*” (European Commission 2019). Furthermore, with respect to the need to act on endocrine disruptors (EDs), stakeholder responses seem to have contributed substantially to the agenda envisaged in the CSS. Such input is certainly important for identifying political areas of concern as well as political issues to act upon, but translating such notions into regulatory action requires careful scientific scrutiny. For example, 61% of the quoted respondents considered themselves less protected from EDs than from other toxic chemicals, such as carcinogenic or mutagenic substances, or substances toxic to reproduction. Scientifically, this is debatable, as most of the currently identified human health-related EDs of concern are in fact carcinogenic and/or toxic to reproduction and are already covered by existing regulations. To be sure, existing concerns in the population have to be taken seriously, but it is the moral and professional obligation of scientific governmental authorities to address such concerns by acting on facts and evidence, clearly distinguishing them from anxieties or beliefs. By putting the latter into perspective, regulatory toxicology can also make a valuable contribution to (EU-wide) risk communication.

Regulatory action based on perceived risks or on biased and flawed assessments is scientifically ill-advised and ultimately has to fail with regard to delivering sustainable solutions. Such an agenda will inherently miss to live up to any promises made. Scientifically this is a situation utterly to avoid, not only for reasons of possible disappointment, or time and effort wasted, but for being able to ultimately deliver on a goal everyone shares—improved protection of public health and the environment.

### A “zero pollution ambition for a toxic-free environment”

The “zero pollution ambition for a toxic-free environment” is at the heart of the CSS and the discussions surrounding it. Notably, precise definitions of the terms “pollution” and “toxic-free” are missing from the CSS. While the COM is certainly aware of the existence of natural toxins and of Paracelsus’ fundamental paradigm (“*All things are poison and nothing is without poison; only the dose makes a thing not a poison*”), the term “toxic-free” nevertheless deserves further attention. The original and slightly more intuitive phrasing used by Goldenman et al. (2017) was “non-toxic”, and they defined a “non-toxic environment” as follows: “*A non-toxic environment should be understood as an environment that is free of chemical pollution*^4^* and of exposures to hazardous chemicals* ***at levels that are harmful to human health and to the environment***. *This target would take into consideration the need to provide vulnerable groups with as much protection as possible, to take account of potential delays between exposure and disease expression, to prevent accumulations of very persistent substances, and to ensure the quality of the material flows foreseen as part of the Circular Economy*.” (Goldenman et al. 2017)

If the new term “toxic-free” were still defined in the same way, then a “zero pollution ambition for a toxic-free environment” refers to an effort to limit the release of dangerous substances to the environment to levels at which they are not harmful, i.e. at which they do not cause damage to human health or the environment. Conversely, (at most) emitting “safe” (non-harmful, non-toxic) levels of dangerous substances to the environment would be consistent with this concept, as in the established risk-centred approach of current chemicals legislation. The concept of “toxic-free” (if interpreted as above) is, therefore, nothing new. In fact, it aligns well with the existing REACH paradigm of “adequately controlled risk”, which already today implies that people should not be exposed to dangerous substances at harmful levels.

In this context, it is trivial yet important to clearly distinguish between hazard, exposure and risk, as only the latter provides information on whether something is harmful (i.e. actually causes harm) or not. Amalgamating the terms “hazard” and “risk” leads to conceptual misunderstandings, with the consequence of fostering muddled conclusions and perceptions. For example, and again trivial to the readers of this journal, the fact that chemicals with hazardous properties can cause harm does not mean that they indeed do so at all doses or by all exposure routes. It would, therefore, be wrong to conclude that just because a chemical has hazardous properties it is a threat to human health (as partly insinuated in the public discussion as well as the CSS and SWDs). If this were so, many of our everyday foods (e.g. coffee, soy products, chocolate, many vegetables, meat, alcohol or sugar) as well as widely used natural substances (e.g. essences, oils, soaps) and chemicals or products (e.g. stainless steel, cosmetics, jewellery) would be deemed inacceptable for use.

From a strict toxicological point of view, there can be no such thing as an inherently safe chemical, as literally every substance, including water, will cause harm if present at the right dose in a particular organ or environment (“*All things are poison*…”). The key to any effective (and efficient) toxicological regulatory action, therefore, necessarily is controlling risk, not hazard. Trying to address questions of chemical safety solely based on the latter would not only often be overprotective, but inherently arbitrary and thus open to scientific and legal challenge. The understanding that chemicals with hazardous properties can in principle be used safely has been one of the cornerstones of modern developed societies, in fact of civilisation as such. In terms of the number of chemicals with hazardous properties contained, e.g. mobile communication devices, personal computers, and cars are “a toxicologist’s nightmare”. But is it scientifically sound to generically assume that these products put people at risk (in terms of chemical safety, and notwithstanding that individual products may)? It is also noted that most, if not all of the technologies needed to bring about the change towards sustainability at the core of the CSS require the use of hazardous chemicals. The list of uses for hazardous chemicals that may be called “essential” by society is, in fact, virtually endless.

### Acting in the face of uncertainty

In the current regulatory system, ideally a significant risk should be demonstrated to justify the need for action. However, this is not always possible due to the lack of available information. There are generally two ways of dealing with this situation. One can (a) generate/gather the information necessary to make a well-founded decision or, where this is not deemed possible or appropriate, (b) refer to precautionary action in spite of the uncertainties. Application of the precautionary principle, however, depends on certain conditions. For instance, principle 15 of the 1992 Rio declaration stated that “…*where there are* ***threats of serious or irreversible damage***, *lack of full scientific certainty shall not be used as a reason for postponing cost-effective measures to prevent environmental degradation*” (United Nations 1992), also underscoring the requirement of proportionality for any regulatory action. In contrast to this statement, the precautionary principle is now frequently called upon in the absence of demonstrated risks. Banning or regulating substances without the need for in-depth assessment would without doubt speed things up tremendously. Moreover, it would be a readily available tool for comforting any hazard-triggered anxieties or unease. Yet, it would also be unscientific, prone to misjudgement and arbitrariness.

The COM itself in their Communication from the year 2000 noted that measures under the precautionary principle should be “*proportional to the chosen level of protection, non-discriminatory in their application, consistent with similar measures already taken, based on an examination of the potential benefits and costs of action or lack of action (including, where appropriate and feasible, an economic cost/benefit analysis), subject to review, in the light of new scientific data, and capable of assigning responsibility for producing the scientific evidence necessary for a more comprehensive risk assessment*.” (European Commission 2000). In contrast, however, the CSS uses precaution on some occasions to justify an envisaged action in the absence of demonstrated risks. Some of these actions have severe consequences, e.g. substances could be banned from consumer products because the “generic approach to risk management” (see “Implications of regulating hazard instead of risk“) is applied, or it is decided to, “*while the generic approach to risk management is not in place, prioritise all the above-listed substances [i.e., carcinogenic, mutagenic, or reprotoxic (CMR) or ED substances] for restrictions for all uses*” (European Commission 2020g, p. 10). However, the CSS does not yet define criteria for deciding on the adequacy, proportionality, and commensurability of such measures and this will be a key task for the COM in the coming months. From a scientific perspective, such criteria cannot be reliable unless rooted in a thorough risk assessment in the first place.

Notably, this is a plea for the responsible use of the precautionary principle, not a plea against its use at all. For example, Art. 1 (3) installs the precautionary principle under REACH and a “generic approach” is already in place (REACH Annex XVII, entries 28–30) to restrict the supply of CMR substances (of CLP Cat. 1) as such or in mixtures to the general public. This appears proportionate, as direct exposure of the general population to CMR substances could otherwise be expected with high likelihood. On the other hand, dangerous substances in consumer articles may in fact be perfectly safe for consumers due to lack of any direct contact and/or negligible migration rates. Arguably, also the identification of Substances of Very High Concern (SVHC) under REACH Title VII constitutes a precautionary, hazard-based regulatory measure obliging registrants to substitute SVHC wherever possible in technical, economic, and societal terms. However, where this is not easily possible, commensurability is achieved by means of a mechanism allowing registrants to obtain an authorisation if they can demonstrate that risks from the substance are adequately controlled (again, the “safe use” paradigm) or that the socio-economic benefit outweighs the expected level of risk.

Regulating chemical hazard instead of risk creates serious problems, which are further discussed in “Implications of regulating hazard instead of risk“.

### Combination effects

A full review of the detailed documentation in the SWDs would go far beyond the scope of this commentary. Hence, this analysis is confined to unintentional/coincidental mixtures, since intentional/foreseeable mixtures are already explicitly addressed in some of the EU’s major regulatory programs (e.g. pesticides and biocides). The application of the science-based concepts used there (Cumulative Assessment Groups (CAGs), Hazard Indices (HI) etc.) could be easily extended to other regulatory domains. Instead, here the implicit claim of the CSS that exposure to unintentional/coincidental chemical mixtures constitutes a major and generic health problem requiring immediate regulatory action, such as the impromptu introduction of a hazard-driven generic MAF (European Commission 2019), shall be investigated.

One of the most prominent references cited by the COM to support their claim of a high and imminent risk of generic mixture toxicity in this context is the report of Goldenman et al. (2017). The CSS quotes this report by stating “*Combined prenatal exposure to several chemicals has led to reduced foetal growth and lower birth rates*”. However, while the original report indeed contains the very same statement, it does not provide data or further evidence to support this claim, which is unfortunate, considering the severity of the decisions derived from it.

Exposure to developmental toxicants during pregnancy is undoubtedly among the highest-ranking regulatory concerns in chemicals regulation; in fact, inter alia the thalidomide crisis of the 1960s was a major driver for establishing a more effective system of drugs as well as chemicals regulation in Europe. However, robust evidence for a wider health concern due to such exposures taking place as a result of a general ineffectiveness of the current regulatory framework is scarce. Most toxicological studies on this issue are rather specific case or proof-of-concept studies (Heise et al. 2018; Knebel et al. 2019; Wittkowski et al. 2019), while epidemiological or biomonitoring studies face inherent conceptual issues with respect to clearly establishing toxicological cause-effect relationships (Tralau et al. 2021).

Scientific progress in analytical chemistry allows an ever more refined and sensitive detection of chemical analytes and biomarkers in environmental and human matrices. This has empowered exposome and (bio)monitoring studies to detect a wide range of substances with higher throughput and at lower levels of detection/quantification (LOD/LOQ) than ever before, allowing for more comprehensive assessments on which substances we are exposed to and to what extent. Importantly, the detection of substances usually does not allow for conclusions regarding their origin or toxicological relevance, whereas targeted analyses are normally only performed retrospectively. Reliable conclusions on increasing or decreasing chemical burden, therefore, require reliable historical controls and references measured under comparable conditions. This severely limits the often-assumed indicative use of such studies, e.g. with regard to conclusions on an increasing toxicological burden due to unintentional/coincidental mixtures. The value of these measurements so far rather lies in the assessment of known and established body burdens and their development over time, e.g. for following the success of implemented regulatory measures. It is thus questionable to which extent such data can currently support the respective statement in the CSS that “*human biomonitoring studies in the EU point to a growing number of different hazardous chemicals in human blood and body tissue, including certain pesticides, biocides, pharmaceuticals, heavy metals, plasticisers and flame retardants*.” (European Commission 2020g, p. 2).

Likewise, the SWD on mixture effects falls short of providing sufficient and well-founded evidence for the CSS’s claims regarding combination effects. Based on two proof-of-concept studies, the SWD makes a principal case for the existence of combination effects as such (European Commission 2020d; Kortenkamp et al. 2009; Kortenkamp and Faust 2018). This being undisputed (and again trivial to the readers of this journal), the subsequent conclusions are difficult to follow [“*Scientific evidence of the strengthened toxicity of* […] *mixtures is mounting* […]. *The total risk related to the exposure to a combination of chemicals typically exceeds the risk related to the exposure to each of the individual chemicals in the mixture on their own, at their respective concentration in the mixture. Therefore, exposure to a mixture can give rise to adverse health and environmental effects, even at levels of exposure which are considered ‘safe’ for the individual chemicals on their own*…”, European Commission (2020d)]. However, the impression that people exposed to mixtures of chemicals will always experience higher toxicity than when exposed to the same chemicals alone is not further supported by the references cited in the SWD. Moreover, it does not reflect the current state of science or that of previous regulatory assessments, e.g. the Scientific Opinion of the COM’s Scientific Committees from 2012 (European Commission 2012). From a regulatory perspective, the latter document actually still subsumes the main points on this issue well:
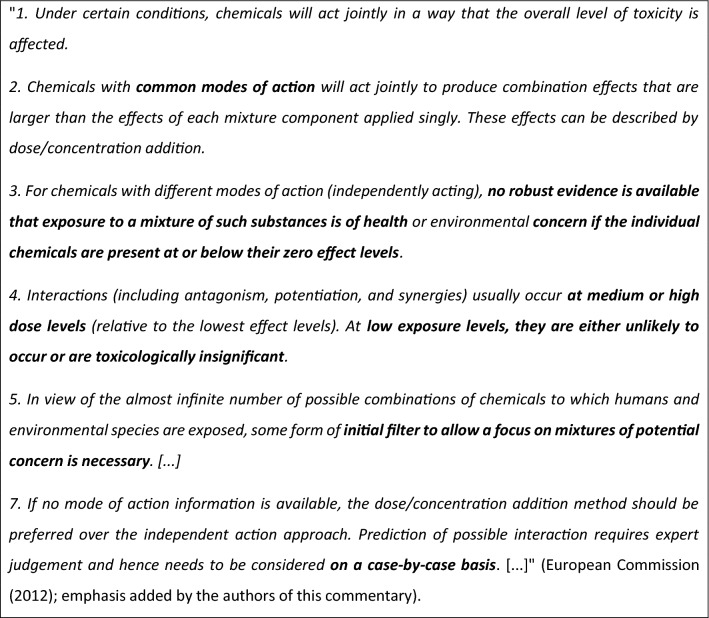


Given that the mechanistic and quantitative requirements for additive (or synergistic/antagonistic) effects are well established, the occurrence of combination effects should not be a default assumption, unless there are data supporting such mechanisms to be potentially relevant. In these cases, recent reviews show that if combination effects occur, they generally concur in size with the prediction made by the dose/concentration addition model rather than exceeding it (Cedergreen 2014; Martin et al. 2021).

Since the publication of the Scientific Committees’ opinion, a number of research projects and publications have looked deeper into the subject, largely confirming their conclusions. Some of these documents are cited by the SWD (Bergman et al. 2019; Bopp et al. 2016; Larsen et al. 2017). These papers (or sources cited within) have repeatedly confirmed that under laboratory conditions chemicals with common/interlinked modes of action (MoAs) can act additively in a mixture. However, this is often achieved using non-standardised or not independently validated in vitro assays, or when theoretically modelling mixture toxicity based on data from the published literature under the assumption of dose/concentration addition (DA/CA). Bergman et al. (2019) report a possible association of concentrations measured in human body fluid samples with an adverse outcome in humans in vivo. However, from the documentation provided [including the underlying publication by Bornehag et al. (2019)], it is impossible to judge the statistical significance of these findings, the influence of potential confounders, or whether true mixture effects, not covered by a risk assessment of the individual mixture components one by one, were present.

To the best knowledge of the authors of this commentary, there is not a single report that would indicate adverse mixture effects due to coincidental mixtures of chemicals present at dose levels below their individual regulatory thresholds to be of wider relevance for humans in vivo. On the other hand, it is acknowledged that providing such data is principally difficult: to convincingly demonstrate this for chronic and long-term effects in a scientifically robust way, resource-intensive prospective epidemiological cohort studies are required. Maybe in the future better data will become available, e.g. from cohorts such as the one described in Kyhl et al. (2015). Moreover, the absence of such studies is of course no proof for the absence of a problem. Yet, in absence of evidence indicating an immediate need for action, the preferred way forward would be to assess the available limited evidence systematically and in an unbiased way. This should then be followed by the initiation of targeted research to close the existing knowledge gaps, as is planned e.g. within upcoming research projects under the Horizon Europe framework, in which also the BfR will be actively engaged.

Toxicologically speaking, for an adverse mixture effect currently not covered by the existing regulatory system to become relevant in terms of regulatory risk assessment, the following aspects need to come together:

1. To act together via DA/CA, chemicals need to have common/interlinked MoAs.
2. The hazard posed by the individual components must be of high concern. This primarily involves substances with CMR properties or Specific Target Organ Toxicity after Repeated Exposure (STOT RE) properties, including those acting via ED-related MoAs.^5^
3. Humans must be exposed to each individual mixture component below their individual regulatory thresholds (otherwise the scenario is already covered by the existing system), and in combination a toxic level must be reached.
4. These levels have to remain more or less constant (not above the individual thresholds, not below an overall toxic level of the mixture) over the whole time-window relevant for the effect (shorter for reproductive effects, longer for STOT RE and cancer).

These preconditions define a very limited and thus accessible chemical space. For the sector of consumer uses under REACH, a rough estimate on the number of substances that could be (but not necessarily are) potentially relevant (as of October 2020, Table 1) was performed.

**Table 1:** Estimated number of substances for which potential adverse combination effects could need to be considered based on REACH registration data

	Number of substances^a^ (% of registered substances)
REACH-registered	23 001 (100)
With expected consumer exposure^b^	7 307 (31.8)
With relevant hazard (CMR 1A/1B/2 incl. Lact., STOT RE 1/2)^c^	2 969 (12.9)
With expected consumer exposure AND relevant hazard classification (CMR 1A/1B/2 incl. Lact., STOT RE 1/2)^c^	924 (4.0)

^a^Data from the ECHA dissemination website Accessed on 05 Oct 2020

^b^Filtered for “consumer use” OR “article service life”

^c^Harmonised or self-classified in any of the listed hazard classes

It is important to understand that no information on MoAs, individual regulatory thresholds or actual exposure levels factored into this analysis, which, therefore, by no means constitutes an estimate of the number of substances actually suspected of causing combination effects. It simply summarises for how many substances it would actually seem worthwhile from a toxicological point of view to take a closer look with respect to MoA and above-threshold exposure. On the other hand, it is acknowledged that this estimate does not cover exposure via the environment or from non-consumer uses. However, for most of the REACH-registered substances levels of consumer exposure from these sources can be estimated to be very low (and at least partially considered by other regulatory sectors already, e.g. in the area of food safety or air quality regulation). Still, some conclusions can be drawn:

- As of October 2020, REACH-registered substances with direct consumer exposure AND relevant hazard made up only 4% of the total number of registered substances.
- Adding the additional requirement that co-exposure with other chemicals needs to occur at levels causing toxicity (only) in combination and constantly so over the relevant time windows, the fraction of substances for which addressing combination effects is relevant will be much smaller in the end. Moreover, in many cases uses in products will already be subject to assessment e.g. by the COM’s Scientific Committees within other, more specific regulatory sectors (cosmetics, food contact materials, toys, etc.).
- Finally, in line with REACH Annex XVII, entries 28–30, consumer uses involving CMR Cat. 1 substances will be confined to products with much less direct exposure, if any, compared to the exposure to neat substances or mixtures.

Altogether, the analysis above, with all its associated uncertainties, seems to hint at a rather low likelihood that EU consumers are currently confronted with significant health risks from the exposure to unintentional/coincidental mixtures. Notwithstanding this general statement, coincidental combinations could of course pose a risk in specific scenarios. For these to be identified, a strong filter is needed to focus available regulatory resources on scenarios of real relevance (Tralau et al. 2021). However, the combination of low incidences and a limited chemical space argues against generic shotgun approaches, such as a hazard-based generic MAF with all its critical implications. There is a real scientific concern that the strong push for such an approach as currently promoted by the COM is not sufficiently data-driven. This is further amplified by the observation that the COM already seems determined to implement this approach before any thorough impact assessment or analysis of the regulatory relevance of this problem has been performed, or alternative options, e.g. those discussed in Rotter et al. (2018), have been explored.

### Endocrine disruptors (EDs)

Same as for potential combination effects, the CSS highlights potential hazards posed by endocrine disrupting chemicals, rather than providing sound evidence that in general potential ED-related risks to human health are not sufficiently covered by the existing regulatory system in the EU. In this case, the associated SWD provides a slightly more differentiated view, noting that “*caution needs to be exercised when using human health and environmental adverse effects as direct and reliable indicators of chemicals policy performance. This is because of the attribution challenge: many of the observed health and environmental adverse effects may derive from multiple causes (life-style, genetics, habitat destruction/degradation, etc.) and it is difficult to determine to what extent exposure to endocrine disrupting substances contributes to the observed adverse effects*.” (European Commission 2020b).

Certain chemicals are able to cause chronic effects mediated via the endocrine system, e.g. reproductive effects, toxicity to specific target organ systems, or cancer, and this is of high regulatory concern. Accordingly, and for a long time already, MS authorities have classified EDs as carcinogenic or toxic to reproduction under the “Dangerous Substance Directive” 67/548/EEC or the CLP Regulation (EC) 1272/2008, and have identified them as SVHC under REACH. Over the last years, clear criteria for the identification of EDs have been installed based on the widely accepted WHO/IPCS definition (Solecki et al. 2017; WHO IPCS 2002) and the OECD Conceptual Framework for Testing and Assessment of Endocrine Disrupting Chemicals (OECD 2018). For the assessment of active substances in biocides and pesticides, guidance (Anderson et al. 2018) is available.

This work undoubtedly needs to be continued. Existing guidance is in continuous need of adaptation. Eventually, scientific progress will show that existing testing or assessment strategies might need to be further fine-tuned or adapted, e.g. with regard to disputed points such as dose-dependency/thresholds, or additional MoAs different from the oestrogen, androgen, thyroid and steroidogenesis (EATS) pathways. Moreover, lack of relevant data on hazard and on underlying ED modes of action constitutes a major problem. Any intention to harmonise and streamline ED identification across different regulatory frameworks is, therefore, highly welcomed and explicitly supported.

Nevertheless, it seems that overall the system established in the EU over the past years allows to effectively identify and regulate industrial chemicals with ED potential, at least where data are available and as far as the assessment of carcinogenic and reprotoxic human health effects is concerned. However, for substances of all tonnage levels registered under REACH mechanistic data are often lacking to identify those candidates in need of clarification with respect to whether their adverse effects are mediated by an ED mode of action. Under REACH, MS authorities have to initiate resource-intensive and highly bureaucratic processes (substance evaluation, SVHC identification) to obtain the necessary information for clarification and to finally corroborate ED identification (cf. also see “Measures proposed by the COM to improve the efficiency of the current system”). These problems are not so much related to the lack of available concepts, but rather to the general—but highly pertinent—problem of data availability. Beyond that, it is, however, not fully clear why the issue is flagged so prominently in the CSS. From a scientific point of view, it also does not seem reasonable to pay more attention to certain CMR substances over other, non-ED CMR substances, just because their underlying MoA involves the endocrine system.

As a prominent measure of the CSS, the COM proposes to introduce additional classification criteria for EDs into the CLP regulation. However, at least as far as the human health sector is concerned, the justification for the urgency, with which this measure is intended to be implemented is unclear, as is its additional benefit for regulatory risk assessment:

- Already today, the available CLP classification criteria cover all major human health-related adverse effects potentially elicited by EDs, e.g. for carcinogenicity and reproductive toxicity. Non-carcinogenic, non-reproductive toxicity effects can be classified as STOT RE, notwithstanding that the latter might need to be further developed in the future to better cover e.g. immunotoxic effects, or with a possible need of adaption of the comparatively high quantitative guidance values currently in place for this criterion.
- Introducing such an additional criterion would also raise the question of whether existing carcinogenicity, reproductive toxicity, and STOT RE entries in Annex VI of the CLP Regulation would need to be revised in retrospect. The resulting additional workload (to be shouldered by MS authorities and ECHA’s Risk Assessment Committee, RAC) would be difficult to justify, given the unclear benefit of the measure.
- Perhaps most importantly, the CLP Regulation implements the United Nations’ Globally Harmonised System (UN GHS) for Classification and Labelling into EU law. Therefore, the normal way to implement a new classification criterion is via the UN GHS. On the other hand, it seems safe to say that an accordingly revised CLP legislation might not be expected before the second half of the decade. Theoretically, the COM could bypass the UN GHS to introduce a new ED criterion, but this would not be a marginal amendment. In fact, it appears questionable whether the UN GHS would survive a unilateral deviation of that dimension in one of its major member regions. And even if it would, the idea of a Globally Harmonised System would be severely compromised by inviting other GHS partners to promote deviations of similar dimension in their own legislative regions. Jeopardising the UN GHS as a whole, however, would be in direct contradiction with the CSS’s declared objective to “*promote, together with industry, the implementation of the Globally Harmonized System of Classification and Labelling of Chemicals (UN GHS) as the means for identifying chemical hazards and communicating them to operators, workers and consumers*” (European Commission 2019).

Seeking other, more efficient and effective—and at the same time, prudent—ways to further advance the needed harmonisation of ED identification and regulation processes across different legislative sectors should, therefore, be considered, carefully weighing regulatory needs and benefits as well as horizontal implications for other regulatory areas and downstream legislation.

## Measures proposed by the COM to improve the efficiency of the current system

### Data availability

Non-compliance by industry with the legislative information requirements constitutes one of the biggest obstacles for identifying substances of concern under REACH. Strikingly, this is not so much a failure of the system itself (the information requirements are in place), but of its proper monitoring and enforcement. It is also a problem of efficiency rather than effectiveness, since missing information can be obtained by established procedures that, however, are both so time- and resource-intensive that they often fail to deliver in practice (cf. also “Time-to-regulation“).

This unsatisfactory situation along with its detrimental effect on risk assessment has been highlighted by MS authorities for years, and its dimension has *inter alia* been characterised by the detailed work of the “REACH Compliance” project (Oertel et al. 2018, 2020; Springer et al. 2015). The outlined initiative for a “zero tolerance approach to non-compliance” (European Commission 2019) is thus highly welcomed. In particular, this initiative raises MS authorities’ hopes for more support to request relevant use and exposure information under REACH in the future. Moreover, not only data availability, but also accessibility should be considered for improving efficiency. As an example, empowering MS to directly access the REACH database with advanced IT-based analysis tools (currently reserved for ECHA) could boost efficiency and at the same time reduce strain on the limited resources of the Agency.

### Time-to-regulation

Shortening the timelines for bringing chemicals under regulation perhaps constitutes one of the most important aspects of improving the efficiency of the current system of chemicals regulation in the EU. One of the major shortcomings of the current system lies in the fact that it normally takes many years from recognising a chemical problem in need of regulation to until that regulation ultimately comes into force. In this context, it is worth pointing out that in most cases the actual scientific risk assessment takes up only a minor fraction of the overall time needed. The major part is spent in time-consuming and bureaucratic procedures, requesting information that already should have been provided upon registration in the first place, or waiting for the next meeting of the regulatory body responsible for the next step in the process.

Oddly, the CSS largely fails to acknowledge this rather low–hanging fruit for improving efficiency, which, in addition, would be in the immediate remit of the COM itself. Instead, the ideas presented strongly focus on risk assessment, one main proposal being the move towards a hazard-based “generic approach to risk management”. Other not-so-new (and in fact, already widely practised) ideas endorsed include the increased use of grouping approaches and the promotion of “one substance, one assessment” (OSOA). Particularly OSOA, however, could become an early victim of the current proposals, given that too little attention is paid to cross-framework compatibility. The latter is a prerequisite for the principle of OSOA to work. Moreover, the MAF as promoted and the proposed classification of EDs under CLP for example only show limited compatibility with the current regulatory frameworks of product safety, cosmetics, plant protection products, biocides or feed and food.

From the perspective of an MS authority, the current processes could be accelerated in many more ways. As one example, the two REACH processes of dossier and substance evaluation could be merged. With both ECHA and the Member States entitled to initiate such work at any point in time, the EU’s Community Rolling Action Plan (CoRAP) could be transformed into a continuous process rather than being updated only once a year, etc.

Accelerating the processes alone will, however, not necessarily procure a higher output with respect to the number of substances regulated per year. In any case, the corresponding regulatory capacity is predominantly limited by the capacity of the EU’s executive regulatory bodies, i.e. ECHA’s committees or the REACH committee hosted by the COM itself. Further streamlining existing processes to reduce the workload per process should, therefore, be aspired and might also be possible to some extent. On the other hand, care needs to be taken that this is not achieved at the expense of scientific quality.

### Implications of regulating hazard instead of risk

Finally, and maybe most importantly, there is the repeatedly noted intention of the CSS to move away from a risk-based to a hazard-based assessment paradigm. As stated in some of the paragraphs before, such a move, tempting as it might be, is bound to create a range of problems and will likely result in a system that by design would be inherently arbitrary and inconsistent:

1. A purely hazard-based regulation does not provide a sound basis for judging the proportionality of the proposed measure.

2. A “generic approach to risk management” (which should rather be termed “generic hazard management”, since a real risk assessment is not involved) will create automatisms resulting in societally undesired consequences. Without proper risk assessment, how to decide whether titanium dioxide, a carcinogen by inhalation in its fine dust form [and now also termed “not safe” for use as food additive by the European Food Safety Authority, EFSA (Younes et al. 2021)], can be safely used in toothpaste or wall paint (Riebeling et al. 2020)? How to decide whether ethanol, a well-known ED, reprotoxicant, and carcinogen by the oral route, can still be safely used for conservation or surface disinfection? Furthermore, as the examples of titanium dioxide or glass and mineral fibres have shown, such automatisms will eventually lead to a situation where the hazard assessment itself is challenged in an attempt to prevent possibly ill-founded, unintended and societally unwanted downstream consequences.

3. Likewise, it is foreseeable that the introduction of a generic, undifferentiated, and solely hazard-based MAF under REACH will lead to a purely calculated risk for many long-established uses (including active substances in pesticides and biocides) that otherwise would be deemed safe. Predictably, this will result in all kinds of unwanted effects, such as:

- a high investment of resources into refining the exposure assessment just to demonstrate that the calculated risk is not likely to be real,
- a high workload for the authorities to define a great number of “essential uses” to be exempted from regulation—in this context, it is again noted that many technologies required to achieve the transition to a green, sustainable and climate-neutral society involve the use of hard-to-replace chemicals associated with hazardous properties;
- unwanted substitution of substances bearing a calculated risk with less well-investigated (but potentially more risky) ones, or
- registrants moving their production outside of the EU to regions with less stringent chemicals control (despite the declared intention of the CSS to create a “level playing field”).

Introducing measures such as the “generic approach to risk management” or a generic MAF—in their proposed generality—will clearly result in a significant quality loss of chemical risk assessment in the EU. It is foreseeable that in the end this will lead to ill-prepared regulatory proposals, the scientific inadequacies of which will eventually have to be dealt with at the “green table” in the REACH committee or in court and might even result in an overall failure of the proposed regulation.

## Conclusion and outlook

Thanks to the existing system of chemicals regulation in the EU, the current level of protection of its population as a whole, including sensitive sub-populations, against chemical risk is among the highest in the world. The rather bleak picture connoted in the CSS and its associated SWDs thus appears misplaced. As a prerequisite for improving public health protection, where necessary, risk assessment methodologies and processes are in constant need of adaption to scientific progress. Nevertheless, a modern and enlightened society should base its decisions on the best scientific knowledge available. In the view of the authors of this strategy, available and well-established indicators of human health quality do not appear to flag a major problem with regard to the general effectiveness of the current system of chemicals legislation in the EU. However, it is fully agreed that the existing regulatory procedures should be accelerated and streamlined, albeit not at the expense of scientific rigour, and that availability and accessibility of the data required for risk assessment still need to be improved significantly. These data, by the way, should actually be available and accessible already based on existing legal requirements. The authors, therefore, are in full support of any initiative towards “zero tolerance on non-compliance”.

Chemicals are a fundamental part of our daily lives, if not to say they are life, literally, as well as evolutionary drivers. More so, they are indispensable building blocks of our society, be it for food production, health protection, amenities, security or as solutions for low-carbon, zero pollution and energy- and resource-efficient technologies, materials and products. It has been a central paradigm of modern societies, supported by long-standing scientific evidence, that not all hazards pose actual risks or, if they do, that these risks can often be adequately controlled. Risk management decisions, therefore, need to be based on risk assessment rooted in the best available scientific knowledge available. Not to endanger this principle, changes to the established cross-sector risk assessment framework, where considered necessary, need to be made with a view to minimising the risk of jeopardising the functioning of that framework as such. In this context, it is a central point of the scientific method that the fundamental hypotheses at the core of newly proposed methodology need to be falsifiable (or verifiable, for that matter). For this reason, the scientific discussion process cannot work on the basis of claims alone; instead, convincing evidence is needed for this purpose. Promoting unjustified beliefs and anxieties for the sake of appeasing public concern in the end bears a substantial risk of amplifying this concern. Without adequately secured expert advice, it will also lead to ill-informed decision-making and ultimately foster the erosion of the authorities’ scientific credibility.

Given the challenges we are currently facing in terms of climate change, sustainable use of industrial and agricultural chemicals, or controlling the Covid-19 pandemic, there is more than ever a need for in-depth discussions on future concepts as well as for regulatory science. It is, therefore, well taken that the COM provides significant funding for research related to the goals of the CSS*,* e.g. under the Horizon 2020/Europe programs or the upcoming Partnership for the Assessmen**t **of Risk from Chemicals (PARC). These projects will take their time, as will the ensuing discussions in the scientific regulatory community. However, this time is needed to safeguard that the “EU Chemicals Strategy for Sustainability” is guided by the best science available. In the view of the authors of this commentary, this will be a fundamental prerequisite for the CSS to become the success story it deserves to become: by creating a sustainable chemicals market, while at the same time continuing to provide a high level of protection for the health of the EU citizens.

## Data Availability

Not applicable.

## Footnotes

1 This term shall be used here to describe whether the system is able to effectively protect the health of the EU population.

2 This term shall be used here to describe whether the system is able to achieve its goals with a minimum of resources.

3 https://eu-rd-platform.jrc.ec.europa.eu/eurocat/eurocat-data/prevalence_en.

4 Notably the term “pollution” itself is, again, not defined in that report.

5 Interestingly, the CSS considers only substances on the SVHC Candidate List or in Annex VI of the CLP Regulation; it is not fully clear, why substances self-classified by the Registrants for such properties should not be included as well.
